# Curvature enhanced NH_2_-MIL-53(Al) electrode for boosting ion diffusion and capacitive deionization defluorination†

**DOI:** 10.1039/d4sc08020c

**Published:** 2025-02-04

**Authors:** Fei Yu, Yidi Yang, Peng Liu, Jie Ma

**Affiliations:** a College of Oceanography and Ecological Science, Shanghai Ocean University No. 999, Huchenghuan Road Shanghai 201306 P. R. China; b Water Resources and Water Environment Engineering Technology Center, Xinjiang Key Laboratory of Engineering Materials and Structural Safety, School of Civil Engineering, Kashi University Kashi 844000 P. R. China jma@tongji.edu.cn +86-2165981629; c Biolin (Shanghai) Trading Company Ltd Rm 1205, Sandhill Plaza, Lane 2290 ZuChongzhi Road, Pudong New District 201203 Shanghai China; d Research Center for Environmental Functional Materials, College of Environmental Science and Engineering, Tongji University 1239 Siping Road Shanghai 200092 P. R. China

## Abstract

Traditional capacitive deionization (CDI) materials typically exhibit low fluorine adsorption capacity (FAC) due to limitations in the optimization of their specific surface area and chemical composition. A prospective strategy for efficient ion storage is modulating the local electric field strength (LEF) by changing the curvature. In this study, we developed a novel modulator-based curvature modulation method to prepare three different morphologies of NH_2_-MIL-53(Al) electrode materials with similar specific surface areas but different curvatures, which were used to investigate the direct constitutive relationship between curvature and CDI performance. The results show that the urchin-like electrode (NCMOF-3) with high surface curvature has an ultra-high fluoride removal capacity (61.29 mg_NaF_ g_electrodes_^−1^), a fast fluoride removal rate (mg_NaF_ g_electrodes_^−1^ min^−1^), and excellent charging/discharging cycle stability (10 000 cycles). CDI performance exceeds all previously reported MOF electrodes. Finally, in combination with the surface curvature/electric field model, we found that higher surface curvature may lead to higher concentration of ion distribution. The mechanism of action may be that high surface curvature enhances the local electric field enhancement (LEFE) effect of the electrode material, which in turn increases the ion storage capacity and diffusion rate during CDI. This study demonstrates firstly the potential effect of curvature on CDI performance by experimental design. More importantly, this study breaks the limitations of material design based on specific surface area and provides new design ideas for next-generation CDI materials based on curvature structure engineering.

## Abstract 2

A curvature modulation method without changing the specific surface area was developed, and the strength of the electric field around the electrode surface significantly affects the ion trapping and diffusion rates during the CDI process.

## Graphical abstract

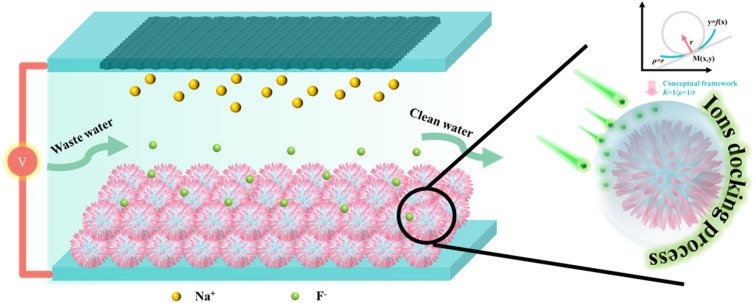

## 1 Introduction

Pollution of freshwater resources seriously affects the quality of drinking water.^1–3^ Fluoride ranks among the most critical and challenging contaminants to eliminate, and excessive intake can cause irreversible damage to teeth and bones, which can be severe enough to lead to permanent bone and joint deformities.^4,5^ As an emerging electrochemical water treatment technology, capacitive deionization (CDI) creates a new pathway for fluoride pollution treatment based on electric double layer (EDL) electrosorption or faradaic reactions.^6–12^ Conventional wisdom suggests that excellent CDI performance can be achieved by optimizing material structural parameters such as specific surface area (SSA) and chemical composition to effectively provide more accessible sites and increase theoretical capacity.^13–18^ However, modulating the pore structure and chemical composition (*e.g.*, heteroatom doping and surface functionalization) can only improve the fluoride ion adsorption capacity (FAC) to a certain extent.^19–21^ For example, doping manganese in porous carbon typically only increases the FAC from <20 mg g^−1^ in undoped carbon to <40 mg g^−1^, probably due to its limited intrinsic SSA and capacity.^22^ Therefore, is there an effective strategy to improve CDI performance that is not limited by the inherent SSA and capacity of the material?

Relevant studies have shown that there is a positive correlation between electric field strength and curvature according to Gauss's law. This effect, also called local electric field enhancement (LEFE),^23^ has been extensively employed in electrochemical sterilization^24^ and electrocatalysis.^25^ So, is it possible to enhance CDI performance by changing the curvature of the materials to control the LEFE effect?

Several recent studies have shown that structural engineering of CDI materials,^26–28^ such as empty tube array engineering^29^ and yolk–shell nanostructures,^30^ can improve CDI performance more than expected, which can be attributed to the LEFE-induced ion docking effect (an effect in which free ions can be temporarily retained by a localized electric field inside the material, analogous to that of a ship that is docked in a harbor undisturbed by the currents of the water^29^) to buffer the cavity with more ions. The tip-shaped structure has also been shown to enhance the local electric field strength and improve the CDI performance.^31^ Xiang *et al.* developed four typical stacking models to further explore possible relationships between curvature, LEFE and CDI performance.^32^ The results show that high curvature materials with enhanced LEFE can significantly improve CDI performance. However, the typical structural materials used in this study had widely varying SSA (118.6 m^2^ g^−1^ for w-ppy, 53 m^2^ g^−1^ for BM-PPy, 25.6 m^2^ g^−1^ for sp-PPy, and 7.7 m^2^ g^−1^ for sh-PPy), and did not exclude the effect of the intrinsic SSA of the materials. Therefore, it remains challenging to rationally design experiments to demonstrate the effect of curvature on CDI performance.

The flexibility of MOF structures is highly adaptable to the design of surface curvature. Inspired by this, based on structural engineering, we developed a curvature modulation method that does not change the SSA of the material. We synthesized three typical morphologies of NH_2_-MIL-53(Al) in block (NCMOF-1), sheet (NCMOF-2) and urchin-like (NCMOF-3). They have similar SSA, pore size distribution and different surface curvatures to verify the effect of curvature structure engineering on CDI performance. As a CDI electrode material, the NCMOF-3 electrode with high surface curvature outperforms all previously reported MOF electrode materials. This includes record high FAC value (61.29 mg_NaF_ g_electrodes_^−1^) and high fluorine removal rate (mg_NaF_ g_electrodes_^−1^ min^−1^), as well as excellent charge/discharge cycling stability (10 000 cycles). More importantly, we excluded the effects of other structural parameters including the intrinsic SSA and pore size distribution of the material, and rationally verified the effects of curvature structure engineering on the ion adsorption capacity and diffusion rate during the CDI process, which provides a new idea for the design of the next-generation CDI materials.

## 2 Materials and methods

### 2.1 Chemicals and materials

Aluminum chlorine hexahydrate (AlCl_3_·6H_2_O) and *N*,*N*-dimethylformamide (DMF) were procured from Sinopharm Chemical Reagent Ltd 2-Amidoterephthalic acid (NH_2_-BDC) was obtained from Adamas. Sodium Fluoride (NaF) and Urea were purchased from Greagent. Ethanol absolute was bought from Lingfeng Chemical Reagent Ltd (Shanghai). These chemicals did not require any other purification procedures before use. 18.2 MΩ cm^−1^ of DI water was used for all experiments.

### 2.2 Synthesis of NH_2_-MIL-53(Al)

The preparation of NH_2_-MIL-53(Al) was based on a previously published method reported with slight differences.^33^ Briefly, 6 mmol of AlCl_3_·6H_2_O was first dissolved in 60 mL of deionized water. Then 6 mmol of NH_2_-BDC was added to the solution with magnetically stirring. Sonicate until the solids are completely dissolved and stirred vigorously for 30 min. Next, add the appropriate amount of urea to the mixed solution and continue stirring for 30 min. The obtained mixtures were then put into a 100 mL PTFE lined autoclave and kept at 150 °C for 5 hours in quiescent conditions. The yellow solid obtained was slowly cooled, collected through centrifugation and several times cleaned with DMF and ethanol absolute. Finally, the creation collected by centrifugation was desiccated at 60 °C under vacuum. The Al-MOF electrodes prepared with different molar masses of urea (0, 13, 18 mmol) were named NCMOF-1, NCMOF-2, NCMOF-3.

### 2.3 Measurements of CDI performance

The electrode material was prepared by scraping the electrode slurry onto graphite paper. The electrode slurry was obtained by having a homogeneous mixture of active materials, carbon black and PVDF in a mass ratio of 8 : 1 : 1. The scratch coated electrodes were then dried at 70 °C overnight. The area of the scratch-coated electrode was 3.5 × 3.5 cm^2^. Activated carbon (AC) electrodes were developed using the synthesis method of Al-MOF electrodes.

The fluoride removal test was conducted in a system incorporating a permanent voltage supply unit (CT3001A, RAND, China), a conductivity analyzer (SevenExcellence S700-K, Mettler Toledo, Switzerland), a percolation flow pump (BT100-2J, LONGE, China), and a CDI cell. CDI cells were used in this study with the same equipment as previously reported. Use NaF to produce F^−^ simulated wastewater, which is then pumped into a CDI cell. During the CDI process, fluorine-containing wastewater was fed into the feed vessel. The change in conductivity of the NaF solution was recorded using a conductivity meter to obtain the concentration of the NaF solution. The volume of solution used in the experiment was 50 mL. The applied voltages were 1.2 V, 1.4 V to 1.6 V and the initial concentration of F^−^ was 100 mg L^−1^. The flow rate was kept at 20 mL min^−1^. The test temperature was kept at 25 °C in all CDI experiments except for the different temperature experiments. The fluorine adsorption capacity (FAC, mg g^−1^) was calculated using eqn (1):1
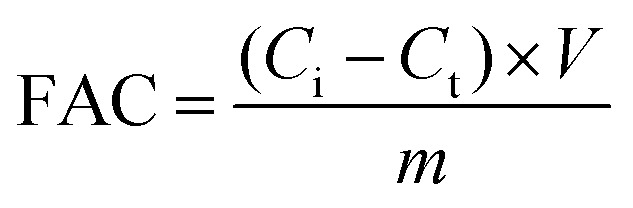
where *C*_i_ (mg L^−1^) is the original concentration of the effluent, *C*_t_ (mg L^−1^) is the experimental treated effluent concentration, *m* (g) is the electrode mass including the Al-MOF electrode and the AC electrode, and *V* (L) is solution volume used in the experiment.

The energy consumption (kW h kg_NaF_^−1^) was obtained from eqn (2):2
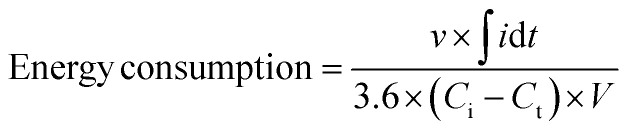
where *v* (V) is the applied voltage in defluorination process, *i* (mA) is the required current in defluorination process, and *t* (s) is the required time in defluorination process.

### 2.4 Electrochemical measurements

For the electrochemical performance test, we prepared 1.0 × 1.0 cm^2^ electrodes for fluoride removal tests. A three-electrode system was used for the experiments, consisting of a working electrode: Al-MOF (WE), a counter electrode: platinum mesh (CE), and a reference electrode: Ag/AgCl (RE). The solvent used for the experiments was 0.5 mol L^−1^ NaF. We performed cyclic voltammetry (CV) and galvanostatic charge discharge (GCD) tests using the Autolab (PGSTAT302N, Metrohm) electrochemical workstation. The frequency range of the electrochemical impedance spectroscopy (EIS) is 0.01 to 10^6^ Hz.

### 2.5 Measurements of quartz crystal microbalance with dissipation (QCM-D)

The QCM-D analysis was performed using a QSense Explorer (Biolin Scientific AB, Sweden). Electrode slurry (slurry composition as prepared for CDI electrodes) was spin-coated on a 5 MHz At-cut QSense gold sensor (QSX301) with an active surface area of 0.79 cm^2^. Tests were performed using an electrochemical module connected to an Autolab electrochemical workstation. The electrolyte used for testing was 10 mmol L^−1^ NaF solution.

### 2.6 Characterizations

The sample morphology was observed using a ZEISS GeminiSEM 300 field emission scanning electron microscope (Germany) and a JEOL JEM-2100Plus high resolution transmission electron microscope (Japan). Samples were characterized for their crystal structures using a Rigaku SmartLab SE X-ray diffractometer (XRD, Japan) under Cu Kα radiation (*λ* = 1.54 Å). The IR adsorption on the samples was registered in the 400–4000 cm^−1^ wave number range using Thermo Scientific Scientific iS20 Fourier Transform Infrared Spectrometer (FTIR, USA). Thermo Scientific K-Alpha XPS spectrophotometer (USA) was used for X-ray photoelectron spectroscopy (XPS) analysis. Surface area analyzer (ASAP 2460, Micromeritics, USA) was used to analyze the N_2_ adsorption/desorption curves at 77 K.

### 2.7 Finite element simulations

**Fig. 1:**
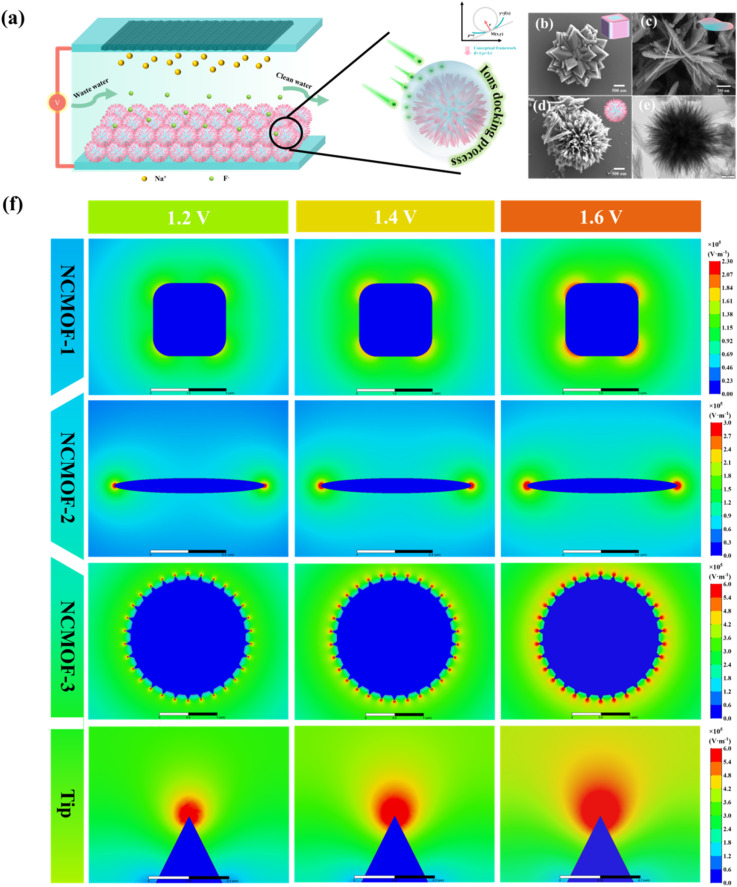
(a) Diagram of the CDI process using urchin-like NCMOF-3 with 3D interconnection network structure as the anode. *K*, *ρ* and *r* denote the curvature, curvature radius and framework radius, respectively. SEM of (b) NCMOF-1, (c) NCMOF-2, and (d) NCMOF-3. (e) TEM of NCMOF-3. (f) Results of simulation for electric field distribution in NCMOF-1 (*ρ* = 1450 nm), NCMOF-2 (*ρ* = 500 nm), NCMOF-3 and the tip of the NCMOF-3 (*ρ* = 50 nm).

The relationship between the electric field near the electrodes and the curvature was simulated by ANSYS Maxwell 2022 software. The simulated geometrical configuration of the electrode is shown in Fig. 1b.

## 3 Results and discussion

### 3.1 Preparation and characterizations

Fig. 1a shows a CDI (fluoride removal) process in an anode based on NCMOF-3 during charging, in which the electrode material loses electrons and its surface is positively charged, which attracts anions in the electrolyte to diffuse along the gradient of the electric field (from low to high potential) until equilibrium is reached, resulting in multiple adsorption modes (surface redox process, EDL, and ion docking process). Therefore, the strength of the electric field around the electrode surface significantly affects the ion trapping and diffusion rates during the CDI process.

For the quantitative analysis in our study, we use the mean Gaussian curvature (*K*_G_) to approximate the surface curvature (*K*) of NCMOF-3 as shown in eqn (3):3
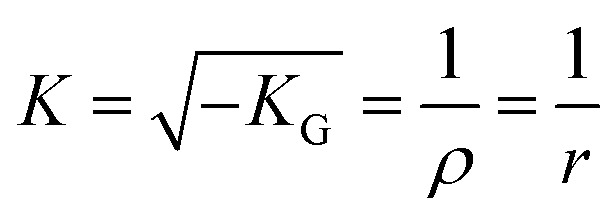
where *ρ* is the curvature radius, and *r* denotes the radius of the curved framework.

To better validate the relations between curvature and CDI performance, we used finite element simulations to investigate the effect of a single structural unit on the induced electric field around the surface of each sample (Fig. 1f), which forms a rapidly decaying electric field gradient from the surface of the sphere (where the intensity is the highest) toward the electrolyte solution. The results show that there is a strong IEFE effect in the localized region of NCMOF-3, and the stronger localized electric field may induce enhanced ion-docking effect, thus improving the CDI performance.

SEM was employed to reveal the microscopic morphology and internal structure within the prepared materials. The morphology for the NH_2_-MIL-53 (Al) changed with urea amount, evolving from a 3D micrometer block to a 3D sea urchin-like shape (Fig. 1b–d). TEM (Fig. 1e) revealed the 3D mesoporous network structure of NCMOF-3 formed by the interconnection of nanosheets. The elemental mapping results of energy dispersive X-ray spectroscopy (EDX) of NCMOF-3 are shown in Fig. S1,† indicating the uniform distribution of the elements on the MOF crystal. Thickness counting analysis of the nanosheets from the SEM images in Fig. S2† showed that the thickness of NCMOF-3 nanosheets ranged from 3–26 nm, which is 10.6 nm on average (Fig. 1d), which is thin *versus* the previously reported thickness range of 35–50 nm of NH_2_-MIL-53(Al) nanosheets. The ultrathin structure facilitates active site exposure and rapid ion transport.

From the N_2_ adsorption–desorption isotherm results on Fig. S3,† it is evident that the isotherm of NCMOF-*x* is of type II with H3-type hysteresis loops, which corresponds to the slit pores formed by the stacking of the laminated structure, which is consistent with the TEM results in Fig. 1b. Three morphologies have similar specific surface areas: 46.0984 m^2^ g^−1^ (NCMOF-1), 51.3661 m^2^ g^−1^ (NCMOF-2) and 49.1347 m^2^ g^−1^ (NCMOF-3). The average pore size of all three morphologies is between 5–8 nm, which is typical of mesoporous materials (usually with a pore size distribution of 2–50 nm) (Table S1†). The existence of massive mesopores facilitates shortening of ion transport paths and accelerates ion diffusion.^7^

From the X-ray diffraction (XRD) results on Fig. S4,† it is evident that the diffractogram of NCMOF-3 matches well with the simulated XRD diffractogram of NH2-MIL-53 (Al) (CCDC No. 901254). There are three sharply defined peaks 2*θ* = 9.2°, 10.0°, and 18.2° belonging to the (110), (200), and (220) crystal planes, respectively, but there is a broadening of the peaks.^34^ This can be attributed to its anisotropic nanosheet morphology and the presence of open metal sites. The nanosheet morphology facilitates open active site exposure, shortens ion transport paths and accelerates ion diffusion. The open metal sites can provide adsorption sites for anion adsorption and facilitate ion storage.

The results of FTIR spectra were shown in Fig. S5,† two peaks at 3493 and 3382 cm^−1^ which belong to hydroxyl telescoping vibration and amino telescoping vibration.^33,35,36^ NH_2_-BDC exhibits a broad vibrational peak disappearance associated with the carboxyl group in the 2500–3300 cm^−1^ range, indicating that the carboxyl group of organic ligand has formed a new chemical bond.^33,36,37^ The new peaks at 1066 and 621 cm^−1^ correspond to Al–O, indicating the presence of coordination between Al and O.^33,36,37^ The presence of amino group would enhance hydrophilicity for Al-MOF and provide more requirements to bind hydrated anions, as well as act as an anion-binding site, thus improving the properties for Al-MOF electrodes.

NCMOF-3 XPS spectra were shown in Fig. S6–10.† The C 1s spectrum (Fig. S6c†) can be divided into three peaks, C–C (284.8 eV), C–N/C–O (286.37 eV) and O–C=O (288.89 eV). As shown in Fig. S8c,† the signal at 399.49 eV of the N 1s spectrum is attributed to the amino group, which can increase the hydrophilicity of the material and at the same time can provide adsorption sites for the formation of hydrogen bonding with F^−^.^38,39^ The O 1s spectrum (Fig. S9c†) can be classified to three peaks near 530.88, 532.18, and 533.91 eV, which corresponds to the Al–O, Al–OH, and H_2_O species, respectively.^40^ The Al–OH and H_2_O substances, which are rich in –OH, contribute to the hydrophilic properties of the material as well as to the adsorption of ions.^41^

### 3.2 Formation mechanism

**Fig. 2:**
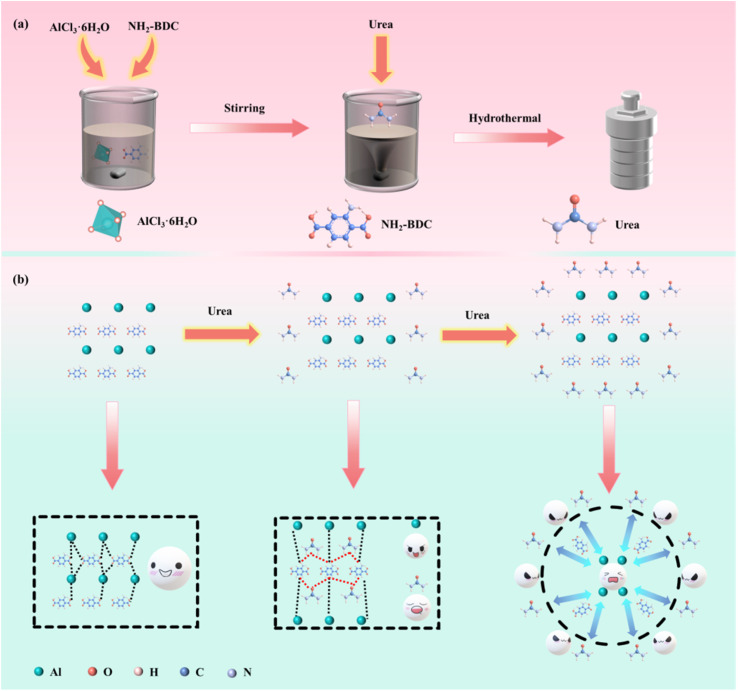
(a) Schematic diagram of the synthesis process of NCMOF-*x*. (b) Diagram of the possible formation mechanism of NCMOF-1, NCMOF-2, NCMOF-3.

Fig. 2a shows the preparation process of NCMOF-*x*. Briefly, we synthesized NCMOF-*x* affected by different concentrations of modulators by hydrothermal method using deionized water as solvent. Fig. 2b shows the possible evolutionary mechanisms for the different morphologies. With increasing amount of urea, NH_2_-MIL-53(Al) exhibits three different morphologies, 3D micrometer blocks, 2D nanosheets and 3D urchin-like. On the one hand, the decomposition rate of urea is accelerated with the increase of temperature, and the decomposition products make the reaction solution form a weakly alkaline system, which accelerates the deprotonation of NH_2_-BDC, and at the same time ensures that the deprotonation products are connected and coordinated with the metal center Al^3+^ to complete the nucleation and growth of crystals.^33^ On the other hand, urea is stabilized at low temperatures, and urea itself can act as both a hydrogen-bond acceptor and a hydrogen-bond donor, competing for hydrogen-bonding sites, thus forming intermolecular hydrogen bonds with NH_2_-BDC, and acting as a “reverse competitive coordination” in the nucleation of organic ligands with metal centers, which effectively inhibits the overgrowth of the MOF crystals in the vertical direction.^42^ This effect leads to anisotropic growth of MOF crystals into 2D nanosheets.^43^ In order to reduce the total system free energy, the nanosheets are intersected at an angle during the growth process to make the lattice orientation uniform, and combined with the Ostwald ripening principle, the final growth is a 3D network structure composed of aligned nanosheets.^44^ This structure not only has ultrathin nanosheets that can shorten ion transport, but also has a large surface curvature that modulates the surface electric field distribution, which can enhance the ion docking effect, thus realizing the rapid and stable trapping of fluoride ions.^45,46^

### 3.3 Electrochemical kinetics performance

**Fig. 3:**
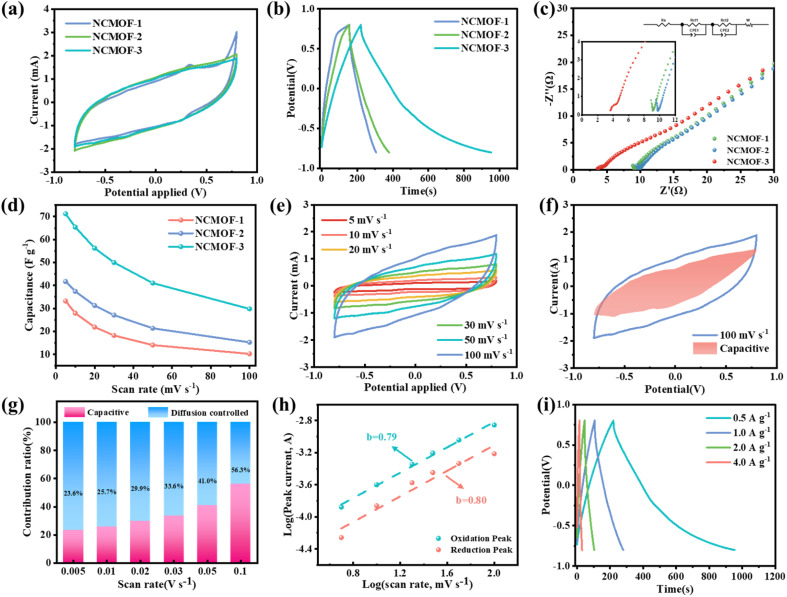
(a) CV curves of the NCMOF-1, NCMOF-2, and NCMOF-3 at a scan rate of 100 mV s^−1^. (b) GCD curves of the NCMOF-1, NCMOF-2, and NCMOF-3 at a current density of 0.5 A g^−1^. (c) EIS of the NCMOF-1, NCMOF-2, and NCMOF-3 electrodes, inset: the equivalent circuit to simulate EIS. (d) Specific capacitances of the NCMOF-1, NCMOF-2, and NCMOF-3 at various scanning rates. (e) CV curves at different scan rates of NCMOF-3 electrode. (f) Total current and capacitive current (shade regions) of NCMOF-3 electrode at 100 mV s^−1^. (g) Normalized ratio of diffusion and capacitive at different scan rates of NCMOF-3 electrode. (h) Power law relationship between peak current and scan rates of NCMOF-3 electrode. (i) GCD at different current densities of NCMOF-3 electrode. Curves.

CV measurements were performed on the resulting material at high sweep rates from 5 to 100 mV s^−1^. The closed region of the CV curve increases with increasing scan rate as shown in Fig. 3e and S11,† which is due to the fact that ions have enough time for diffusing toward the surface of the electrode under low scan rates. In addition, the polarization of NCMOF-2 and NCMOF-3 disappeared at +0.8 V at a scan rate of 100 mV s^−1^ (Fig. 3a), indicating that the addition of the moderator enlarged the voltage interval of the Al-MOF electrodes, and the ultrathin nanosheet structure shortened the ionic/electronic diffusion paths, which avoided the ionic buildup on the surface of the electrodes, and improved the electrochemical performance of the Al-MOF electrodes. The specific capacitance of NCMOF-3 is the largest at different scanning speeds (Fig. 3d), which may be related to the smaller radius of curvature of NCMOF-3. The enhanced surface electric field distribution favors the ion docking effect, increases the ion adsorption capacity, and exhibits a higher capacitance.

For further identifying the outstanding fluoride removal performance of NCMOF-3 electrode, the capacitive contribution and diffusion control contribution of NCMOF-3 electrode were analyzed. The ratio of diffusion capacitance was lower than 50% at scan rates up to 100 mV s^−1^, as shown in Fig. 3g. Fig. 3f shows the capacitance contribution of the NCMOF-3 electrode at a sweep rate of 100 mV s^−1^, which corresponds to the 56.3% share of the capacitance at 100 mV s^−1^ in Fig. 3g, illustrating that the electrode material combines the capacitance and diffusion-controlled properties, which can be beneficial to meet the requirements of capacitive deionization for high fluoride removal rate and high fluoride removal capacity at the same time.^47–49^ The capacitance contribution of NCMOF-3 electrode was increased with increasing scanning rate, which proved that NCMOF-3 electrode has a good ability to remove fluorine. Fig. 3h reveals the peak current *versus* scan rate after logarithmic transformation as a linear correlation, which explains electrochemically stored ion behavior for the electrode materials. *b* values can be obtained from the slope coefficient. Usually, the *b* value is between 0.5 and 1. Low *b* values (close to 0.5) indicate diffusion-controlled behavior, while high *b* values (close to 1) indicate capacitive behavior.^50^ In contrast, where *b* value is in the range between ≈0.5 and 1, the pseudocapacitive process is used as a primary mechanism for storage of electrode materials. The pseudocapacitive behavior of NCMOF-3 electrodes with *b* values between 0.79 and 0.80 greatly enhances their fluorine storage capacity.

GCD measurements were carried out under different currently densities ranging from 0.5 to 4.0 A g^−1^ (Fig. 3i and S12†). The curves exhibited similar triangles, with the time of discharge decreasing as current density increased. Lower current densities result in longer charging and discharging times for the electrodes, which is due to adequate electron diffusion under low current densities. If current density is excessively low, the charging and discharging capabilities of the electrodes are reduced. Apparently, the NCMOF-3 electrode exhibits the longest discharge time (Fig. 3b) and therefore has the highest capacitance, which is consistent with the CV results. The fractionally symmetric and marginally warped curves also show the pseudocapacitive organization of NCMOF-3 electrodes. As shown in Fig. 3c, the Nyquist plots indicate that the NCMOF-3 electrodes have the lowest resistance, which is attributed to the ion-docking effect induced by the unique morphology as well as the fast diffusion of ions/electrons on the nanosheets, and its unique structure compensates for the poor electrical conductivity of the MOFs to a certain extent.

### 3.4 Defluorination performance

**Fig. 4:**
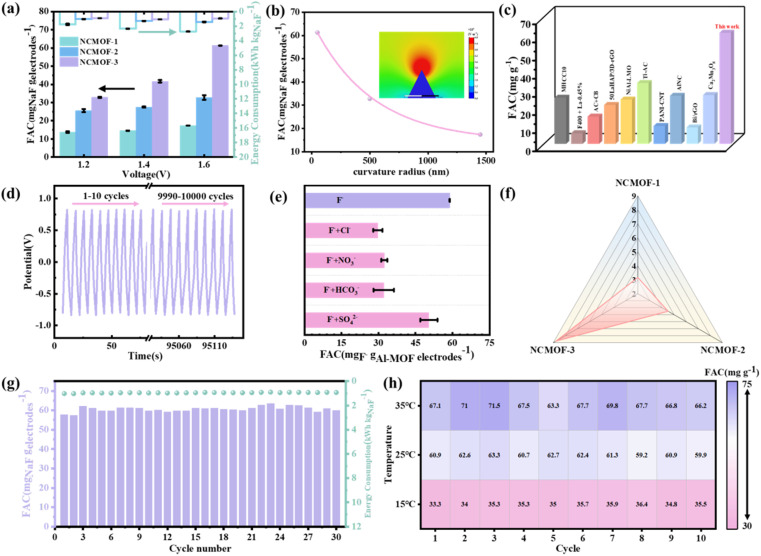
(a) FAC and energy consumption during defluorination of NCMOF-1, NCMOF-2, NCMOF-3 electrodes in a 100 mg L^−1^ F^−^ (NaF) solution with a flow rate of 20 mL min^−1^. (b) Fitted relationship between fluoride removal capacity and radius of curvature. The inset of (b) shows a color map of the electric field distribution on the surface of a urchin-like model. (c) Comparison of fluoride removal performance with different electrode materials in the literature. (d) 10 000 GCD cycle curves of NCMOF-3 electrode in the first 10 cycles and the last 10 cycles at 10 A g^−1^. (e) Fluorine removal performance of NCMOF-3 in the presence of foreign ions. (f) Fluoride removal rates of NCMOF-1, NCMOF-2 and NCMOF-3. (g) The long cycle of fluoride removal. (h) The fluorine removal performance of NCMOF-3 electrode under different temperature conditions.

First, the CDI performance of Al-MOF electrodes with different morphologies for fluoride ion removal was evaluated (Fig. 4a). It can be found that the fluoride removal capacities of NCMOF-2 and NCMOF-3 were 32.74 mg_NaF_ g_electrodes_^−1^ and 61.29 mg_NaF_ g_electrodes_^−1^, respectively, which were significantly higher than that of NCMOF-1 (17.38 mg_NaF_ g_electrodes_^−1^). The micrometer blocks are unfavorable for ion storage and it is difficult for ions to enter the interior of the blocks, while nanosheets benefit from exposing active sites and shortening ion transfer distances. To further evaluate the effect of morphology on the fluoride removal performance, we further compared and analyzed the energy consumption and fluoride removal rate of the three electrodes, and unsurprisingly, NCMOF-3 had the lowest energy consumption (0.94 kW h kg_NaF_^−1^) as well as the fastest fluoride removal rate (8.78 mg_NaF_ g_electrodes_^−1^ min^−1^). The order of magnitude of the fluoride removal rate of NCMOF-x was (Fig. 4f): NCMOF-3 (8.78 mg_NaF_ g_electrodes_^−1^ min^−1^) > NCMOF-2 (4.51 mg_NaF_ g_electrodes_^−1^ min^−1^) > NCMOF-1 (3.16 mg_NaF_ g_electrodes_^−1^ min^−1^). This can be attributed to the smaller radius of curvature to optimize the surface electric field distribution and the enhanced ion docking effect to strengthen the interfacial mass transfer of fluoride ions for fast and efficient fluoride ion capture. Quantitatively, the relationship between the fluoride removal capacity and the curvature radius can be fitted (Fig. 4b), and an exponential polynomial function, eqn (4), can be obtained, which can be expressed as:

4 FAC = *A*_1_e^−*ρ*/*a*^_1_ + *A*_2_e^−*ρ*/*a*^_2_ + *A*_3_e^−*ρ*/*a*^_3_ + *b*

where FAC is the fluoride removal capacity, *ρ* denotes the curvature radius, and *A*_*n*_, *a*_*n*_ (*n* = 1, 2, 3) and *b* are constants (Table S2†).

We further compared the NCMOF-3 electrode with other materials in the literature for its excellent fluoride removal performance (Fig. 4c), which is an exciting result and a new breakthrough in fluoride removal performance for our group. The above results indicate that the ion docking effect is conducive to the enhancement of the CDI performance of the electrode, and the NCMOF-3 electrode can achieve both high defluorination capacity and low energy consumption in high fluoride wastewater, which is conducive to its popularization and application.

We further evaluated the practical application potential of the NCMOF-3 electrode. The fluoride removal capacity and energy consumption of the NCMOF-3 electrode for 30 long cycles of fluoride removal at 1.6 V are shown in Fig. 4g. After 30 adsorption–desorption cycles, the defluorination capacity of NCMOF-3 electrode still maintains more than 95% defluorination capacity, showing excellent defluorination performance and excellent cycle stability. We also performed a GCD long-cycle test of the NCMOF-3 electrode. The NCMOF-3 electrode underwent 10 000 charge/discharge cycles under 10 A g^−1^ current density (Fig. 4d), and there was no obvious degradation during the cycling process, which indicates that the electrode has excellent durability, which is consistent with the results of the fluoride removal long-cycle. In order to further verify the excellent stability of the NCMOF-3 electrode under different environmental conditions, it was tested for 10 cycles of fluoride removal under different temperature states, and the results are shown in Fig. 4h. It can be found that the NCMOF-3 electrode still maintains high defluorination capacity and excellent cycle stability under high temperature environment (35 °C). The fluoride removal capacity was reduced in the low-temperature environment (15 °C), which may be related to the slow ion transport kinetics at low temperatures,^51^ but NCMOF-3 still showed excellent cycling stability. The above results fully demonstrate the excellent cycling stability of the NCMOF-3 electrode under different temperature conditions, and show its potential application under different ambient temperature conditions.

We further simulated the effects of different common competitively adsorbed anions (Cl^−^, SO_4_^2−^, HCO_3_^−^ or NO_3_^−^) on the NCMOF-3 electrode in real water (Fig. 4e). Measurements were made at a primary concentration at 100 mg L^−1^, 1.6 V. 1 : 1 molar ratio of F^−^ to competing adsorbed anions. It is evident that the capacity of the NCMOF-3 electrode for F^−^ adsorption decreases in existence of other anions due to competition of other anions with active adsorption sites. The influence of each anion to F^−^ adsorption order by Cl^−^ > HCO_3_^−^ > NO_3_^−^ > SO_4_^2−^. It can be found that the removal ability of chloride ions on fluoride ions is significantly more effective, primarily caused by chlorine radical of 0.332 nm, smaller than the hydration radius of fluoride ions of 0.352 nm, which is conducive to the entry of Cl^−^ into electrode and competing for fluoride ions adsorption sites.^52^ Sulfate ions have a hydration radius of 0.379 nm and therefore have less effect on fluoride ion removal. Bicarbonate has a negative effect on the removal of fluorine from the NCMOF-3 electrode. Previous studies showed that bicarbonate causes scaling of electrodes, which affects its performance in removing fluoride.^53^ Although the fluorine removal performance of the NCMOF-3 electrode was interfered by coexisting ions, it still had a fluorine removal capacity of not less than 30 mg g^−1^, which is higher than most of the materials that have been reported, further illustrating the potential of the NCMOF-3 electrode for practical applications.

### 3.5 Removal mechanisms of F ions

**Fig. 5:**
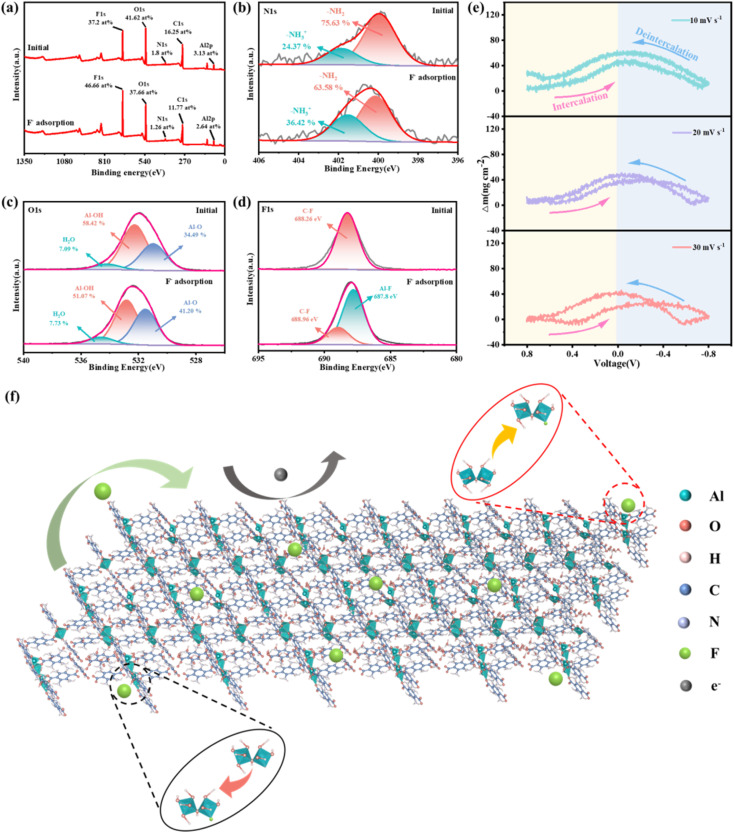
(a) XPS survey spectrum, (b) N 1s, and (c) O 1s of NCMOF-3 before and after adsorption. (d) High-resolution F 1s XPS spectra of NCMOF-3 after adsorption. (e) △*m* of NCMOF-3 electrode from EQCM-D during CV at different scan rates. (f) Diagram of the adsorption mechanism of F^−^.

The CDI defluorination mechanism of NCMOF-3 was explored by the first analysis using ectopic XPS. It was obvious that the F 1s in the electrode increased from 37.2 at% to 46.66 at% after F^−^ adsorption by NCMOF-3 (Fig. 5a), which indicated that F^−^ was successfully adsorbed onto the surface of NCMOF-3. Fine spectral analysis can further explain the evolution mechanism of F^−^ on NCMOF-3. In the N 1s spectrum of Fig. 5b, the peak area of –NH_2_ decreased from 75.63% to 63.58% and that of –NH_3_^+^ increased from 24.37% to 36.42% after adsorption of F^−^. Due to the presence of free H^+^ in water, NCMOF-3 can be protonated by binding additional H^+^ at the surface. As a result, these edge sites (–NH_3_^+^) can adsorb F^−^ through electrostatic interactions.^54^ In addition, combined with the full spectrum analysis, the peak area of the O 1s fine spectrum decreased from 41.62% to 37.66% after the adsorption of F^−^ by NCMOF-3, suggesting that the adsorption of F^−^ also consumed chemisorbed oxygen on the electrode surface.^55^ In the O 1s spectrum of Fig. 5c, the peak area of Al–OH decreased from 58.42% to 51.07% after adsorption of F^−^, whereas the peak area of Al–O increased from 34.49% to 41.2% and the peak area of H_2_O increased from 7.09% to 7.73%. It indicates that the abundant hydroxyl group (–OH) in NCMOF-3 is mainly replaced by fluoride through ligand exchange reaction.^56^ In the F 1s spectrum of Fig. 5d, the adsorption of F^−^ resulted in the formation of a new Al–F bond, which was due to the electron transfer between the open metal sites at the edges of the 2D nanosheets and F resulting in the adsorption of ions.^57^ The adsorption mechanism is shown in Fig. 5e. The above factors work together to realize the excellent fluoride removal performance of NCMOF-3.

We have used EQCM-D to realize *in situ* monitoring of mass changes at NCMOF-3 electrodes during electrochemical processes. The mass change can explain the mechanism of ion embedding and de-embedding on the electrode, providing strong support for its practical application. As shown in Fig. 5e, the mass change of the NCMOF-3 electrode during the CV process can be observed. The mass of the electrode showed good cyclic variation at all three scan rates (10, 20, and 30 mV s^−1^), and when charging, the mass of the electrode increased, indicating that due to the adsorption of fluoride ions on the NCMOF-3 electrode. On the contrary, when discharging, the mass of the electrode decreases due to the desorption of fluoride ions, and when the discharge ends, the mass of the electrode returns to the initial value. This indicates that the effects of ion embedding and detachment on the structure of NCMOF-3 electrodes are reversible and further demonstrates the stability of NCMOF-3 electrode.^41,58^

## 4 Conclusions

In summary, we verified the effect of curvature on CDI performance. The reasonableness of the simulated surface curvature/electric field model was investigated by using MOF as a typical model material, excluding the effects of specific surface area and pore structure. Theoretical and experimental results show that high surface curvature significantly enhances the CDI performance by optimizing the electrostatic field and ion diffusion paths. As a CDI electrode material, NCMOF-3 electrodes with high surface curvature outperform all previously reported MOF electrode materials. This includes record high FAC (61.29 mg_NaF_ g_electrodes_^−1^) and high fluorine removal (8.78 mg_NaF_ g_electrodes_^−1^ min^−1^), as well as excellent charge/discharge cycle stability (10 000 cycles). In this study, a general strategy to enhance the IEFE effect through curvature structure engineering to improve the performance of CDI was validated through rational design experiments, which provides new ideas for the design of next-generation CDI materials.

## Data availability

The data that support the findings of this study are available from the corresponding author upon reasonable request.

## Author contributions

Fei Yu: supervision and writing (original draft, review and editing). Yidi Yang: conceptualization, data curation/analyses, investigation, methodology, validation, visualization, and writing (original draft). Peng Liu: use of software. Jie Ma: funding acquisition, project administration, resources, supervision, and writing (review and editing).

## Conflicts of interest

The authors declare no conflict of interest.

## Supplementary Material

SC-016-D4SC08020C-s001
