# Supplementary material for: Curvature enhanced NH2-MIL-53(Al) electrode for boosting ion diffusion and capacitive deionization defluorination
Source: Chem Sci. 2025 Feb 4;16(11):4635–45. doi: 10.1039/d4sc08020c (PMC11808401; doi:10.1039/d4sc08020c)
Supplement: SC-016-D4SC08020C-s001 [file SC-016-D4SC08020C-s001.pdf]

Supplementary Information (SI) for Chemical Science.
This journal is © The Royal Society of Chemistry 2025

Supporting Information

Curvature Enhanced NH_2-MIL-53(Al) Electrode for Boosting Ion Diffusion and Capacitive Deionization Defluorination

Fei Yu[1,2], Yidi Yang[1], Peng Liu[3], Jie Ma[2,4*]

[1] College of Oceanography and Ecological Science, Shanghai Ocean University, No 999, Huchenghuan Road, Shanghai, 201306, P. R. China

[2] Water Resources and Water Environment Engineering Technology Center, Xinjiang Key Laboratory of Engineering Materials and Structural Safety, School of Civil Engineering, Kashi University, Kashi 844000, P. R. China

[3] Biolin (Shanghai) Trading Company Ltd, Rm 1205, Sandhill Plaza, Lane 2290 ZuChongzhi Road, Pudong New District, 201203 Shanghai China

[4] Research Center for Environmental Functional Materials, College of Environmental Science and Engineering, Tongji University, 1239 Siping Road, Shanghai, 200092, P. R. China

Corresponding author:

Jie Ma, Telephone: +86-02165981629, Email: jma@tongji.edu.cn

Table of Contents

1. Supplementary Figures 3

Figure S1. EDS mapping of NCMOF-3. 3

Figure S2. Thickness distribution of NCMOF-3 measured by SEM images. 4

Figure S3. N_2 adsorption/desorption curves of NCMOF-1, NCMOF-2, and NCMOF-3. 5

Figure S4. XRD of NCMOF-1, NCMOF-2 and NCMOF-3. 6

Figure S5. FTIR of NCMOF-1, NCMOF-2 and NCMOF-3. 7

Figure S6. Survey XPS spectrum of (a) NCMOF-1, (b) NCMOF-2, and (c) NCMOF-3. 8

Figure S7. XPS spectra for C1s of (a) NCMOF-1, (b) NCMOF-2, and (c) NCMOF-3. .9

Figure S8. XPS spectra for N1s of (a) NCMOF-1, (b) NCMOF-2, and (c) NCMOF-3. 10

Figure S9. XPS spectra for O1s of (a) NCMOF-1, (b) NCMOF-2, and (c) NCMOF-3. 11

Figure S10. XPS spectra for Al2p of (a) NCMOF-1, (b) NCMOF-2, and (c) NCMOF-3. 12

Figure S11. CV curves of (a) NCMOF-1, and (b) NCMOF-2, at scan rates from 5, 10, 20, 30, 50 to 100 mV s^{-1}. 13

Figure S12. GVD curves of (a) NCMOF-0, and (b) NCMOF-1, at current densities from 0.5, 1.0, 2.0, to 4.0 A g^{-1} 14

2. Supplementary Tables 15

Table S1. Specific surface area and Average pore diameter of NCMOF-1, NCMOF-2, and NCMOF-3. 15

Table S2. The values of the parameters in equation (4). 16

1. Supplementary Figures

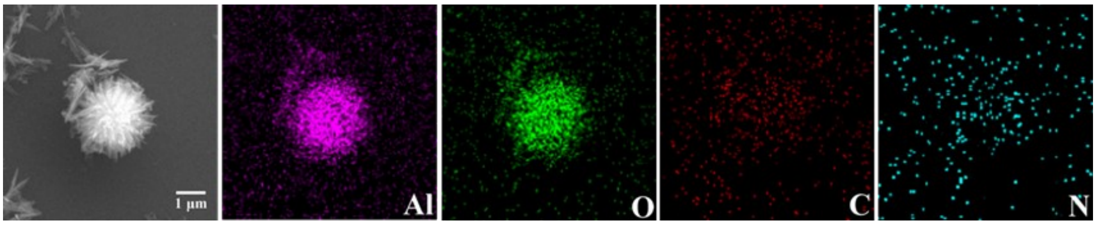

Figure S1. EDS mapping of NCMOF-3.

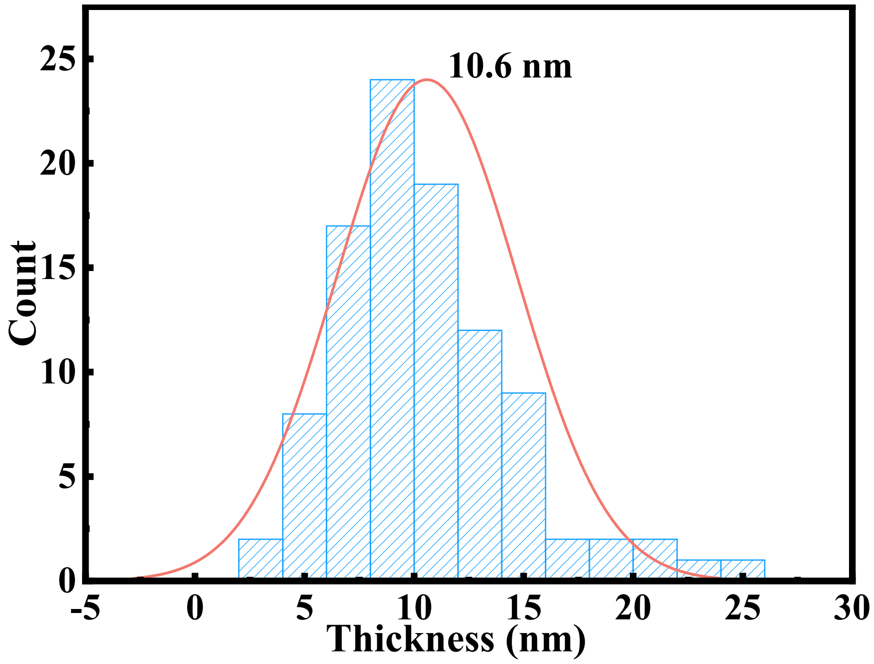

Figure S2. Thickness distribution of NCMOF-3 measured by SEM images.

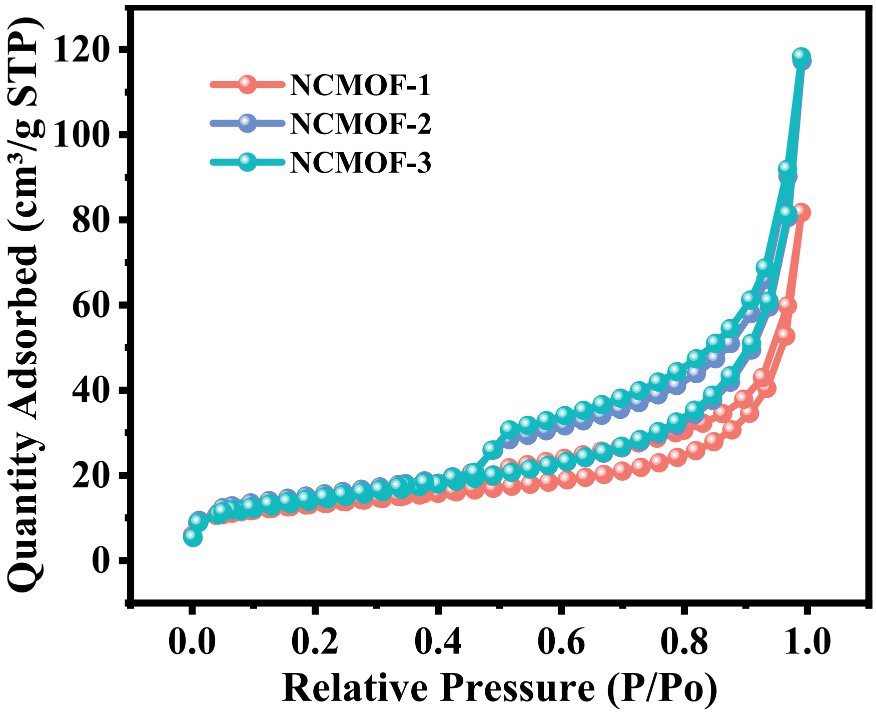

Figure S3. N_2 adsorption/desorption curves of NCMOF-1, NCMOF-2, and NCMOF-3.

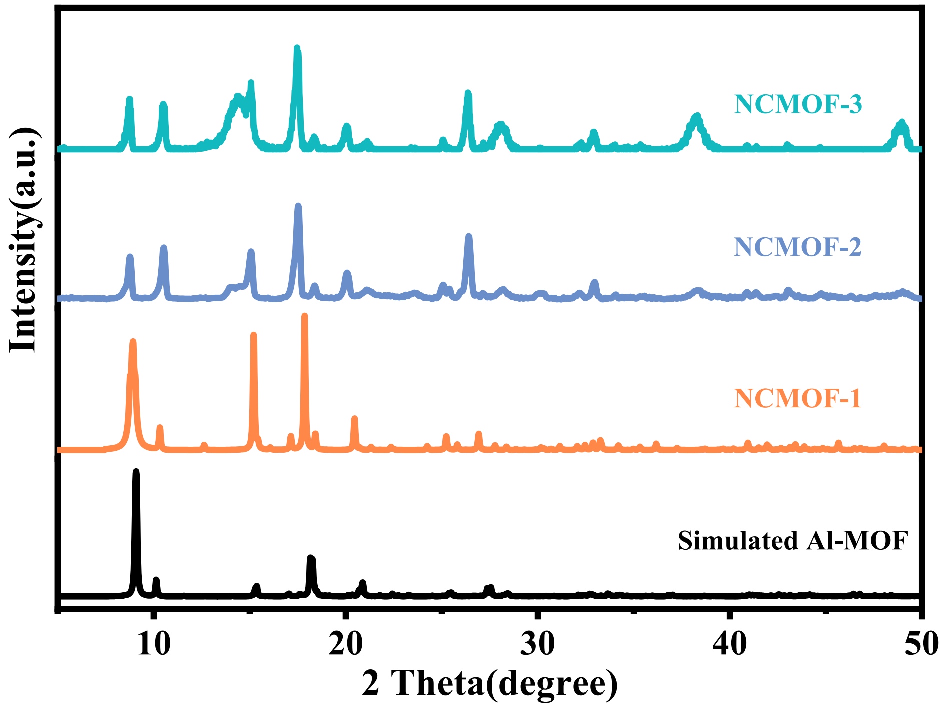

Figure S4. XRD of NCMOF-1, NCMOF-2 and NCMOF-3.

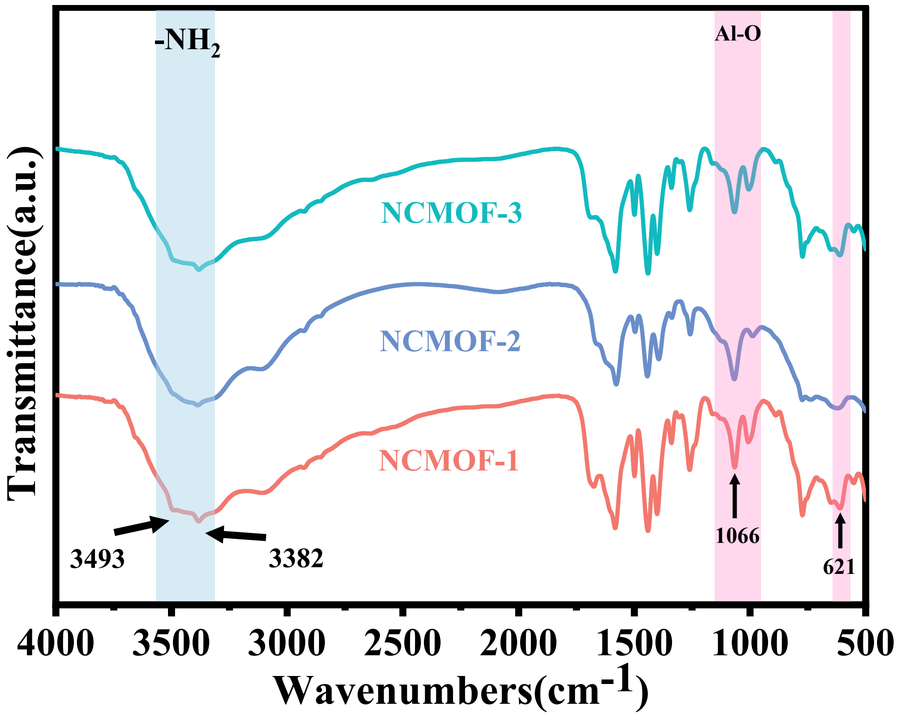

Figure S5. FTIR of NCMOF-1, NCMOF-2 and NCMOF-3.

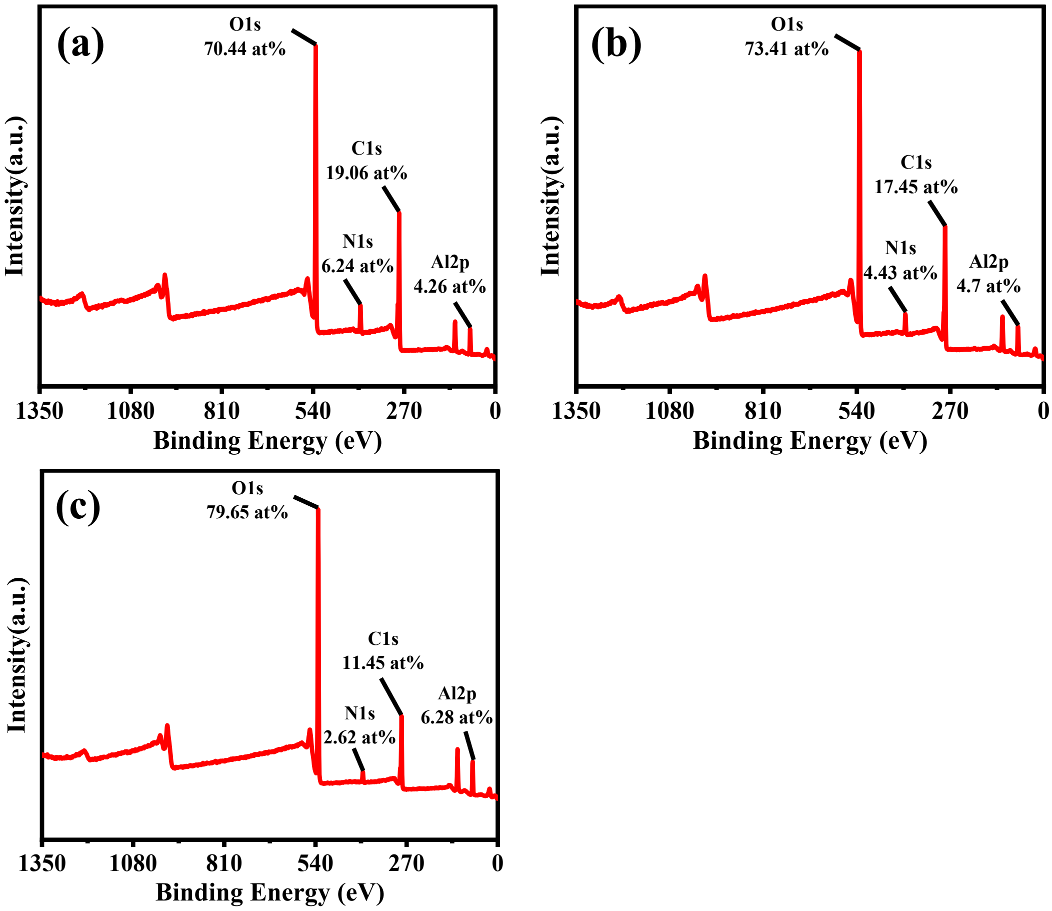

Figure S6. Survey XPS spectrum of (a) NCMOF-1, (b) NCMOF-2, and (c) NCMOF-3.

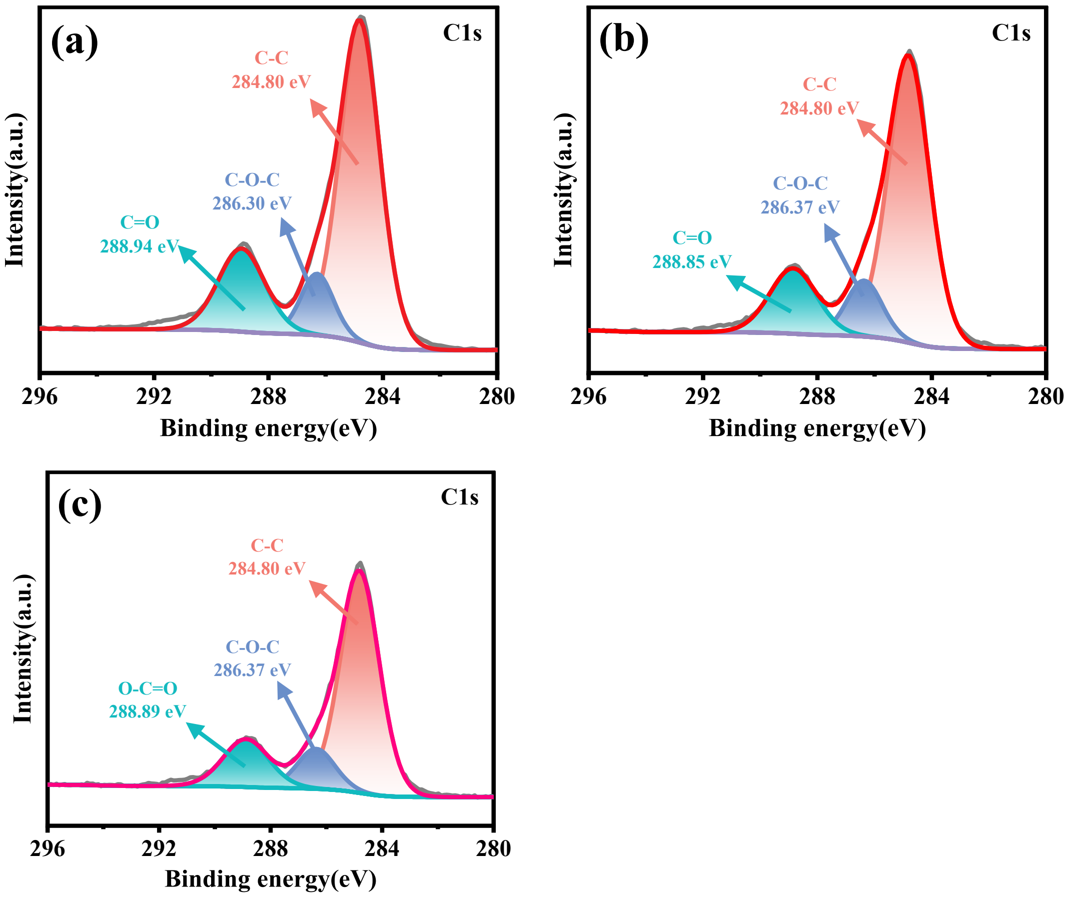

Figure S7. XPS spectra for C1s of (a) NCMOF-1, (b) NCMOF-2, and (c) NCMOF-3.

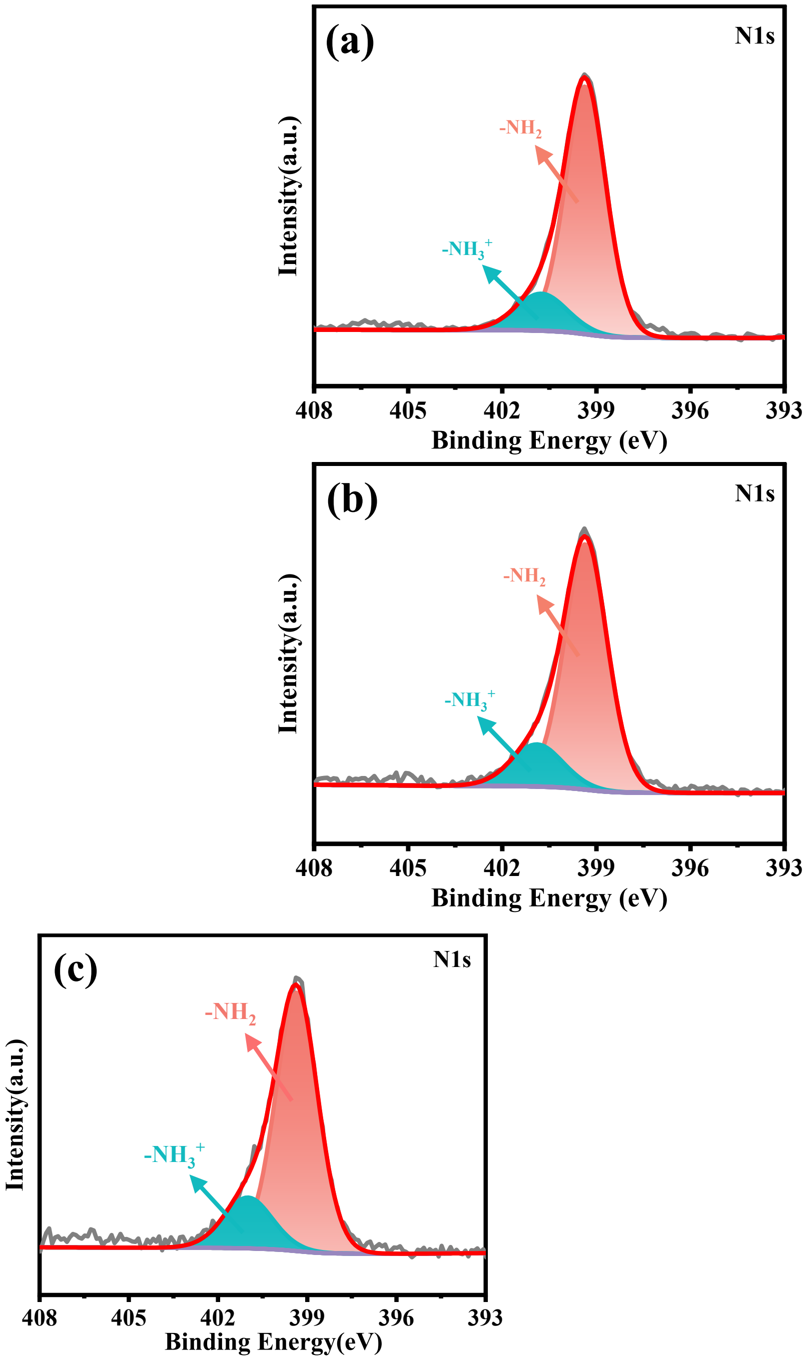

Figure S8. XPS spectra for N1s of (a) NCMOF-1, (b) NCMOF-2, and (c) NCMOF-3.

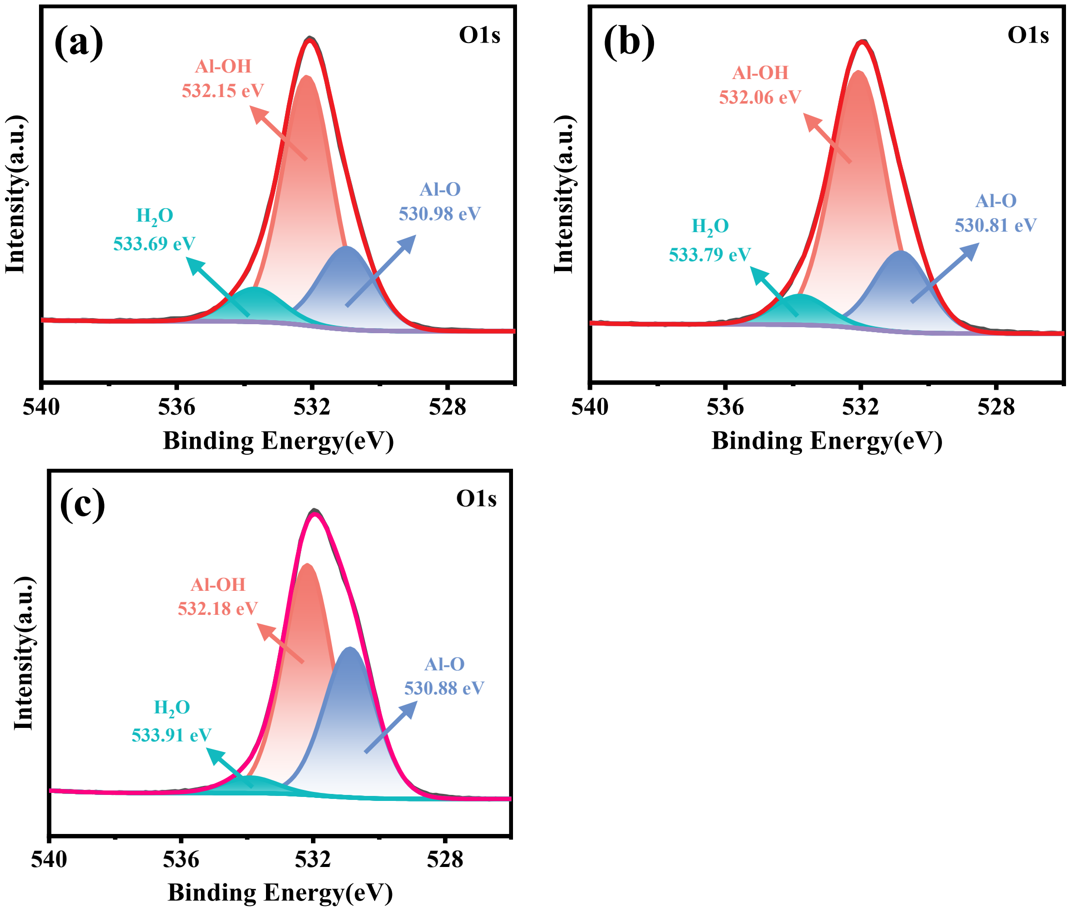

Figure S9. XPS spectra for O1s of (a) NCMOF-1, (b) NCMOF-2, and (c) NCMOF-3.

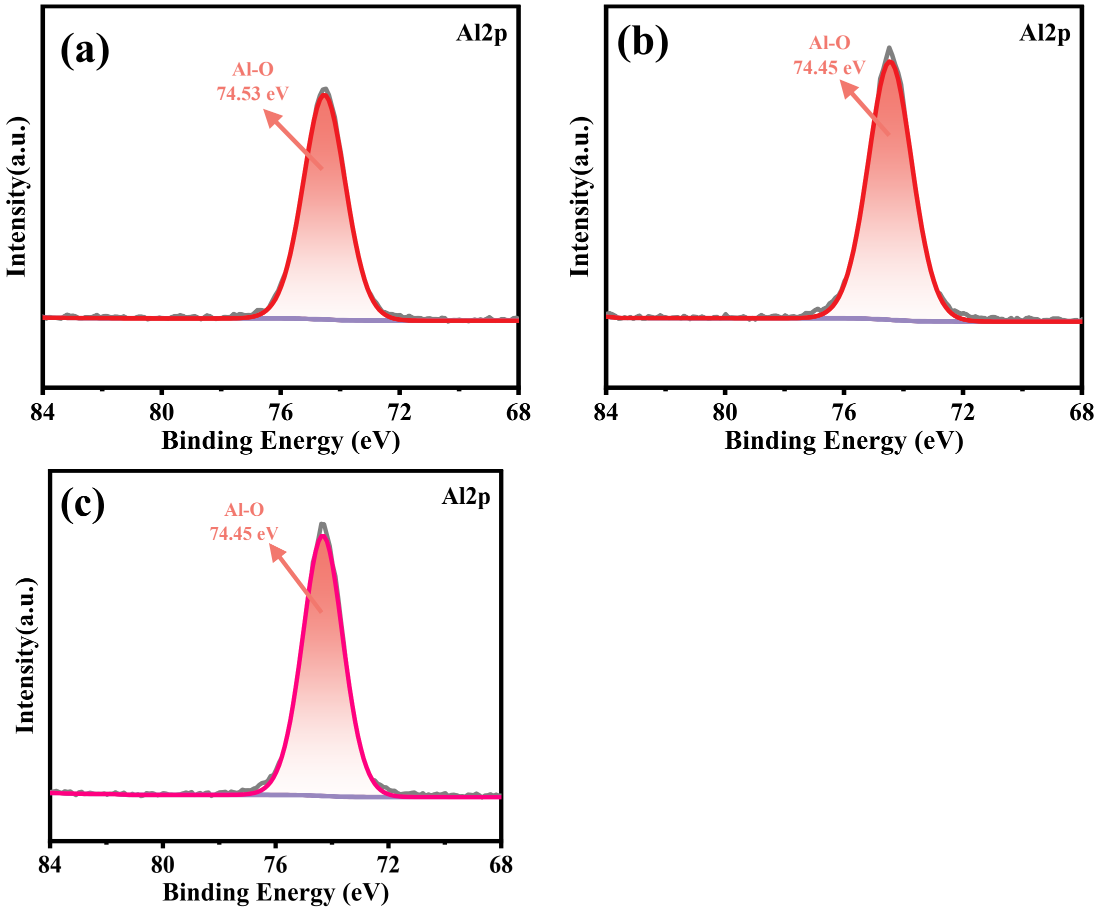

Figure S10. XPS spectra for Al2p of (a) NCMOF-1, (b) NCMOF-2, and (c) NCMOF-3.

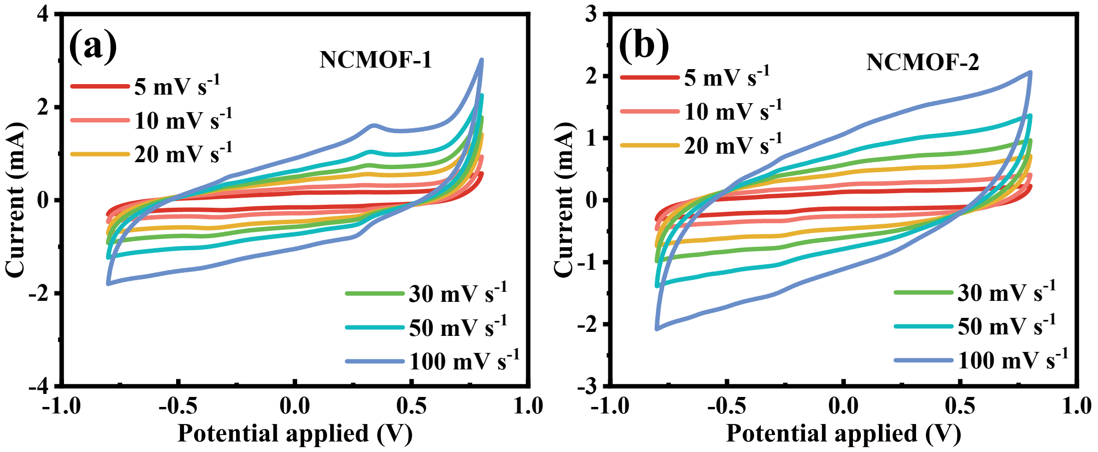

Figure S11. CV curves of (a) NCMOF-1, and (b) NCMOF-2, at scan rates from 5, 10, 20, 30, 50 to 100 mV s^{-1}.

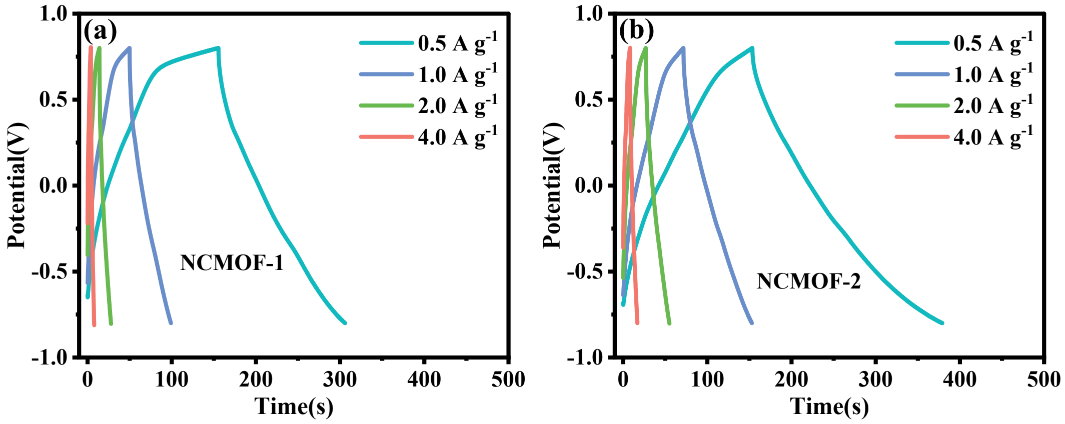

Figure S12. GVD curves of (a) NCMOF-0, and (b) NCMOF-1, at current densities from 0.5, 1.0, 2.0, to 4.0 A g^{-1}.

2. Supplementary Tables

Table S1. Specific surface area and Average pore diameter of NCMOF-1, NCMOF-2, and NCMOF-3.

Sample	Specific surface area S_{BET} ($m^2\ g^{-1}$)	Average pore diameter (nm)
NCMOF-1	46.0984 $m^2\ g^{-1}$	5.1344 nm
NCMOF-2	51.3661 $m^2\ g^{-1}$	6.7467 nm
NCMOF-3	49.1347 $m^2\ g^{-1}$	7.4828 nm

Table S2. The values of the parameters in equation (4).

Parameters	A_1	a_1	A_2	a_2	A_3	a_3	b	R^2
Value	17.25	460.84	17.26	512.04	17.26	563.25	14.33	0.99894
